# Successful resection of a neuroendocrine tumor in the gallbladder: a case report

**DOI:** 10.1186/s40792-020-01055-w

**Published:** 2020-10-27

**Authors:** Hideo Tomihara, Kazuhiko Hashimoto, Tomoko Wakasa, Hajime Ishikawa, Tomoyuki Tsujimoto, Atsushi Gakuhara, Shuichi Fukuda, Katsuya Ohta, Kotaro Kitani, Jin-ichi Hida, Yoshio Ohta, Masao Yukawa

**Affiliations:** 1 Department of Surgery, Kindai University Nara Hospital, Otoda-cho 1248-1, Ikoma, Nara 630-0293 Japan; 2 Department of Pathology and Laboratory, Kindai University Nara Hospital, Otoda-cho 1248-1, Ikoma, Nara 630-0293 Japan

**Keywords:** Gallbladder, Neuroendocrine tumor, Carcinoid

## Abstract

**Background:**

Gallbladder neuroendocrine tumors (GB-NETs) are extremely rare, representing only 0.5% of all NETs because no neuroectodermal cells are present in the gallbladder. In 2019, the World Health Organization updated the classification of NETs based on their molecular differences. The mutation status of *DAXX* and *ATRX* has been added to the criteria for well-differentiated NETs.

**Case presentation:**

A 50-year-old man presented to our hospital for further examination of a gallbladder polyp. He had no right quadrant pain, fever, jaundice, weight loss, or carcinoid syndrome-related symptoms. The patient hoped to avoid cholecystectomy. During the 3-year observation period, the polyp gradually increased in size from 8.3 to 9.9 mm. He decided to undergo surgery, and whole cholecystectomy was successfully performed. Immunohistochemical staining revealed positivity for chromogranin A, synaptophysin, and CD56. The Ki-67 index was < 3%. Taken together, these results led to a diagnosis of a grade 1 GB-NET. We also performed immunohistochemical staining of DAXX and ATRX, which revealed that DAXX protein expression was negative. The patient’s postoperative course was uneventful, and he developed no recurrence for 8 years after surgery.

**Conclusion:**

We experienced a very rare case of GB-NET. Obtaining a correct preoperative diagnosis is quite difficult at the first evaluation. A GB-NET should be considered as a differential diagnosis of gallbladder tumors.

## Background

A neuroendocrine tumor (NET) is a rare type of tumor with an incidence of about 5.25 per 100,000 people [1]. It has been reported to occur in various organs including the lungs, thyroid, ileum, and pancreas. Gallbladder NETs (GB-NETs) are extremely rare, representing only 0.5% of all NETs [2] because no neuroectodermal cells are present in the gallbladder. Multipotent stem cells or neuroendocrine cells involved in intestinal or gastric metaplasia of the gallbladder epithelium have recently been considered the origin of GB-NETs [3]. Most GB-NETs are incidentally diagnosed after cholecystectomy for acute cholecystitis, chronic cholecystitis, or other suspected biliary diseases without specific symptoms, while the frequency of carcinoid syndrome is only < 1% [4, 5]. We herein report our experience with a very rare case of a GB-NET characterized by a typical clinical presentation, nonspecific imaging findings, and typical immunohistochemistry findings.

## Case presentation

**Fig. 1:**
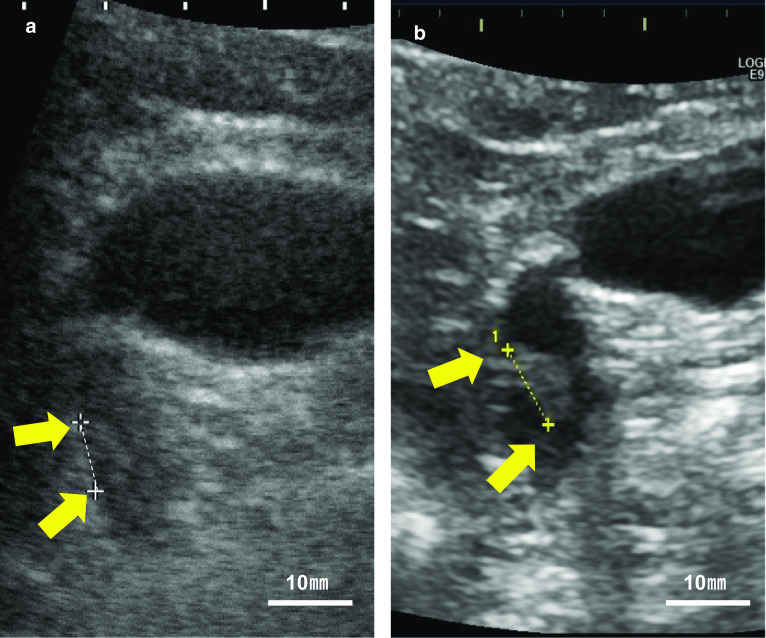
Ultrasound of the gallbladder showed a well-defined, smooth mass of mixed hypo- and iso-echogenicity at the gallbladder neck (arrows). **a** The diameter of the mass was 8.3 mm. **b** The tumor increased in size to 9.9 mm. Scale bar = 10 mm

**Fig. 2:**
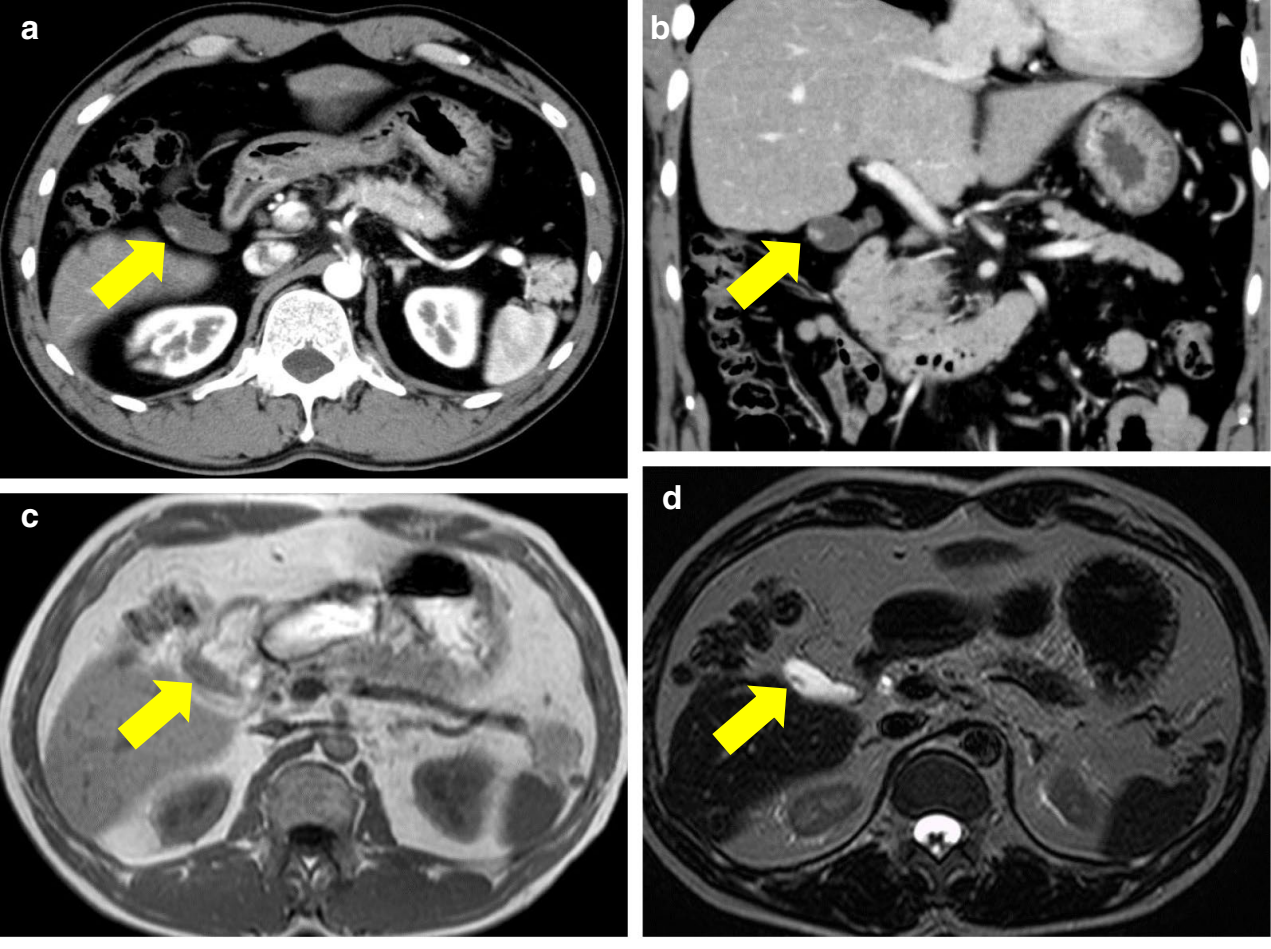
Contrast-enhanced computed tomography of the abdomen showed an enhanced polyp in the **a** axial and **b** coronal sections. **c** Axial T1-weighted magnetic resonance imaging showed that the polyp was isointense. **d** T2-weighted magnetic resonance imaging showed that the polyp was of low intensity

A 50-year-old man with a history of an atrial septal defect and hypertension presented to our hospital for further examination of a previously diagnosed gallbladder polyp. He had no right quadrant pain, fever, jaundice, weight loss, or carcinoid syndrome-related symptoms such as diarrhea, flushing, edema, or wheezing. The patient hoped to avoid cholecystectomy. Abdominal ultrasonography revealed an 8.3-mm elevated polyp in the region around the gallbladder neck (Fig. 1a). During the next 3 years, the polyp gradually increased in size to 9.9 mm (Fig. 1b). Furthermore, contrast-enhanced computed tomography showed enhancement of the polyp (Fig. 2a, b). Magnetic resonance cholangiopancreatography (MRCP) showed no abnormality in the bile duct or pancreatic duct, while the polyp showed iso-intensity on T1-weighted images (Fig. 2c) and low intensity on T2-weighted images (Fig. 2d).

**Fig. 3:**
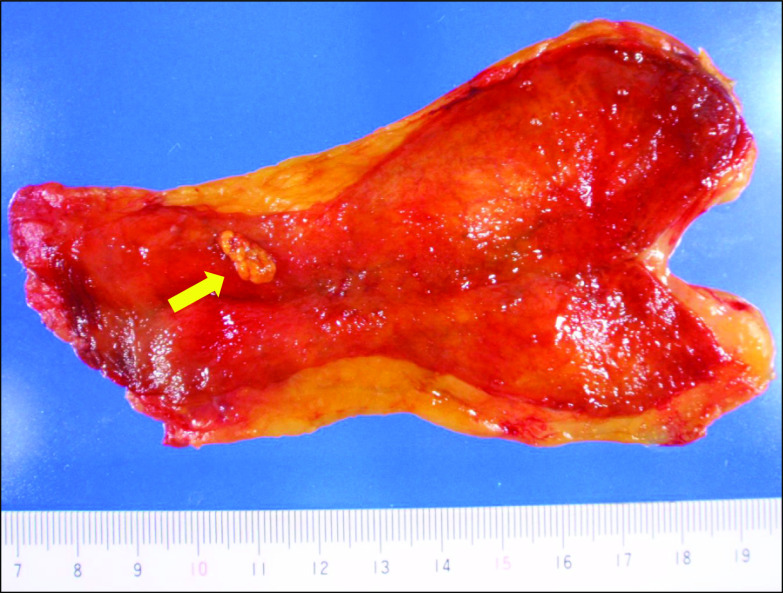
Examination of the resected specimen revealed a 10-mm polyp in the gallbladder neck

**Fig. 4:**
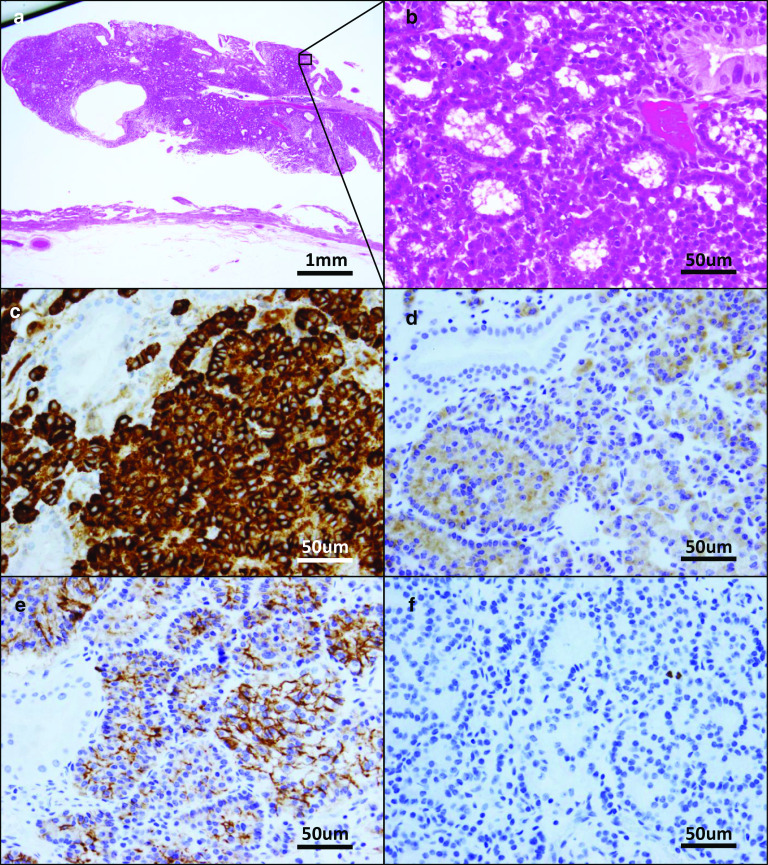
Definitive diagnosis of a neuroendocrine tumor of the gallbladder relies on the pathological results. **a**, **b** Hematoxylin and eosin staining of the gallbladder tumor. Immunohistochemical staining showed positivity for **c** chromogranin A, **d** synaptophysin, and **e** CD56. **f** The Ki-67 index was < 3%

**Fig. 5:**
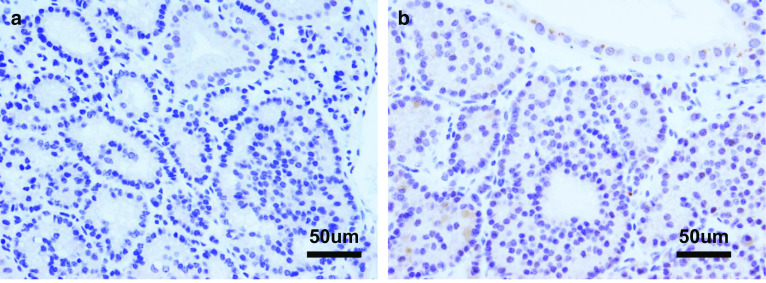
Immunohistochemistry for **a** DAXX and **b** ATRX

The patient had smoked 20 cigarettes per day for 20 years. He also had a history of drinking alcohol (one bottle of beer per day). Physical examination showed no abnormalities. Blood tests also showed no abnormalities, including elevations of tumor markers such as carcinoembryonic antigen (1.5 ng/ml) and carbohydrate antigen 19–9 (9.3 U/ml). Taken together, these results suggested that the polyp included a malignant component. Therefore, we performed laparoscopic cholecystectomy. The whole gallbladder was successfully removed. Macroscopic examination of the resected specimen revealed a tumor of approximately 10 mm in diameter in the gallbladder neck region (Fig. 3). Histologically, hematoxylin and eosin staining showed an alveolar pattern consisting of monomorphous round cells with centrally located nuclei (Fig. 4a, b). The extent of tumor infiltration was within the lamina propria of the mucosal membrane, and the tumor resection margin was negative. Immunohistochemical staining revealed positivity for chromogranin A, synaptophysin, and CD56 (Fig. 4c–e). Immunohistochemical staining of Ki-67 showed that the proliferative index in the tumor was < 3% and that the mitotic count ranged from 0 to 2 per 10 high-power fields (Fig. 4f). Taken together, these results led to a diagnosis of a grade 1 well-differentiated NET. Immunohistochemical staining was negative for DAXX and slightly positive for ATRX (Fig. 5a, b). The patient’s postoperative course was uneventful, and he developed no signs of recurrence either clinically or radiologically for 8 years.

## Conclusions

The term “carcinoid” has historically been confusing. In 1907, “karzinoide” (carcinoid) was defined as a benign tumor by Oberndorfer [6], but a population of such tumors has been recognized as a type of malignancy because they have the ability to metastasize mainly to the liver. Thus, in the last 20 years the definition of carcinoid has changed to NET. In 2000, the World Health Organization (WHO) classification of digestive system tumors divided NETs into three types: well-differentiated endocrine tumor, well-differentiated endocrine carcinoma, and poorly differentiated endocrine carcinoma/small-cell carcinoma [7]. In 2010, the WHO updated the classification of NETs into three new categories according to the proliferative ability of the tumor: grade 1, 2, and 3 NETs [8]. More recently, in 2019, the WHO updated the classification of NETs based on their molecular differences: well-differentiated NETs are defined by mutations in *MEN1*, *DAXX*, and *ATRX*, whereas neuroendocrine carcinomas usually have *TP53* or *RB1* mutations. We have herein reported a very rare case of a GB-NET with immunohistochemical staining of DAXX and ATRX [9].

NETs are reportedly rare; they arise mainly in the gastrointestinal tract, where they account for < 2% of all primary gastrointestinal tumors [2]. Primary NETs in the gastrointestinal tract occur most frequently in the rectum, small intestine, pancreas, stomach, colon, duodenum, and appendix. Among them, GB-NETs are extremely rare, representing 0.04% to 0.20% of all NETs [4, 5]. This rarity is explained by the fact that no neuroectodermal cells are present in the gallbladder. Multipotent stem cells or neuroendocrine cells involved in intestinal or gastric metaplasia of the gallbladder epithelium have been considered the origin of GB-NETs [3]. Preoperative diagnosis of GB-NETs is difficult when patients have no specific symptoms and radiological findings are not different from those of other gallbladder tumors. GB-NETs are often incidentally detected by routine histological examination of specimens after cholecystectomy with a preoperative diagnosis of acute or chronic cholecystitis or suspected malignancy with a gallbladder polyp [10–14]. Ayabe et al. [15] analyzed 754 patients with GB-NETs using the National Cancer Database in the United States. They reported that patients were predominantly female (*n* = 518, 69%) and white (*n* = 503, 67%) and presented with stage IV disease (*n* = 295, 39%) and high-grade lesions (*n* = 312, 41%). Sixty-four percent of the patients underwent surgery, mainly simple cholecystectomy (*n* = 480, 64%), and 145 patients (21%) underwent multimodal therapy. The median overall survival of the patients with GB-NETs was 25 months. The authors reported that older age, large cell histology, and positive margins were independently associated with worse overall survival. Cen et al. [16] analyzed 248 patients with GB-NETs using the Surveillance, Epidemiology, and End Results (SEER) database. They reported that most patients with GB-NETs were women (67.3%), white (77.0%), and married (61.7%). Most tumors were < 2 cm in size (31.0%), stage G3 (25.8%), and distant SEER stage (41.1%). Patients who underwent gallbladder surgery had significantly better survival. The authors also reported that older age, an unmarried status, large tumor size (> 5 cm), and distant SEER stage were significant independent predictors of worse survival. In our case, the patient had no symptoms. Contrast-enhanced computed tomography revealed that the polyp in the gallbladder was enhanced and had been gradually enlarging. At this point, we suspected a malignancy of the gallbladder and performed whole cholecystectomy. Immunohistochemical staining of chromogranin A, synaptophysin, and CD56 suggested that the tumor was a GB-NET [15–17]. Immunohistochemical staining of Ki-67 revealed that the tumor was a grade 1 well-differentiated NET [8]. Compared with previous reports, our patient was an Asian man with a < 1.0-cm grade 1 well-differentiated NET and had long survival of > 8 years after successful total cholecystectomy. GB-NETs should be considered among the differential diagnoses of gallbladder tumors.

Furthermore, we were able to stain serial tumor specimens with antibodies to DAXX and ATRX, the mutation status of which has been added to the criteria of well-differentiated NETs [9]. In our case, DAXX protein expression was negative, as expected, whereas ATRX expression was slightly positive. Indeed, there is some discrepancy between gene expression levels and functional protein levels, but it is curious that the tumor was negative for DAXX staining.

The serum chromogranin A and urinary 5-HIAA concentrations have been the gold standard biomarkers for the identification and follow-up of carcinoids [15]. Although their specificity is very high (close to 100%), their sensitivity is very low. In the near future, noninvasive modalities such as liquid biopsy of the gene mutation status of *MEN1*, *DAXX*, and *ATRX* could be an entirely new strategy to diagnose gastrointestinal well-differentiated NETs.

We have herein reported a rare case of a GB-NET. Obtaining a correct preoperative diagnosis is quite difficult at the first evaluation of such patients. As indicated by our case, a GB-NET should be considered as a differential diagnosis of gallbladder tumors.

## Data Availability

Not applicable.

## Abbreviations

NET: Neuroendocrine tumor
GB-NET: Gallbladder neuroendocrine tumor
WHO: World Health Organization
SEER: Surveillance, Epidemiology, and End Results

## References

[1] Yao JC, Hassan M, Phan A, Dagohoy C, Leary C, Mares JE (2008). One hundred years after "carcinoid": epidemiology of and prognostic factors for neuroendocrine tumors in 35,825 cases in the United States. J Clin Oncol.

[2] Eltawil KM, Gustafsson BI, Kidd M, Modlin IM (2010). Neuroendocrine tumors of the gallbladder: an evaluation and reassessment of management strategy. J Clin Gastroenterol.

[3] Monier A, Saloum N, Szmigielski W, Alrashid A, Napaki SM (2015). Neuroendocrine tumor of the gallbladder. Polish J Radiol.

[4] Sanders RJ, Axtell HK (1964). Carcinoids of the gastrointestinal tract. Surg Gynecol Obstetr.

[5] Godwin JD (1975). Carcinoid tumors. An analysis of 2,837 cases. Cancer.

[6] Oberndorfer S (1907). Karzinoide tumoren des dünndarms. Frankfurter Zeitschrift für Pathologie.

[7] Klöppel G, Perren A, Heitz PU (2004). The gastroenteropancreatic neuroendocrine cell system and its tumors: the WHO classification. Ann N Y Acad Sci.

[8] Bosman FT, Carneiro F, Hruban RH, Theise ND (2010). WHO classification of tumours of the digestive system.

[9] Nagtegaal ID, Odze RD, Klimstra D, Paradis V, Rugge M, Schirmacher P (2020). The 2019 WHO classification of tumours of the digestive system. Histopathology.

[10] Soga J (2003). Primary endocrinomas (carcinoids and variant neoplasms) of the gallbladder. A statistical evaluation of 138 reported cases. J Exp Clin Cancer Res..

[11] Modlin IM, Lye KD, Kidd M (2003). A 5-decade analysis of 13,715 carcinoid tumors. Cancer.

[12] Modlin IM, Shapiro MD, Kidd M (2005). An analysis of rare carcinoid tumors: clarifying these clinical conundrums. World J Surg.

[13] Anjaneyulu V, Shankar-Swarnalatha G, Rao SC (2007). Carcinoid tumor of the gall bladder. Ann Diagn Pathol.

[14] Geo SK, Harikumar R, Kumar S, Kumar B, Gopinath A (2007). Gall bladder carcinoid: a case report and review of literature. Trop Gastroenterol.

[15] Ramage JK, Davies AH, Ardill J, Bax N, Caplin M, Grossman A (2005). Guidelines for the management of gastroenteropancreatic neuroendocrine (including carcinoid) tumours. Gut.

[16] Ramage JK, Ahmed A, Ardill J, Bax N, Breen DJ, Caplin ME (2012). Guidelines for the management of gastroenteropancreatic neuroendocrine (including carcinoid) tumours (NETs). Gut.

[17] Klimstra DS, Modlin IR, Coppola D, Lloyd RV, Suster S (2010). The pathologic classification of neuroendocrine tumors: a review of nomenclature, grading, and staging systems. Pancreas.

